# Comments to “PRevention of INCisional hernia after liver transplantation (PRINC trial): study protocol for a randomized controlled trial.”

**DOI:** 10.1186/s13063-020-4053-5

**Published:** 2020-02-11

**Authors:** Janusz Maciej Strzelczyk

**Affiliations:** Uniwersytet Medyczny w Lodzi, Łódź, Poland

**Keywords:** Prophylactic mesh, Beta-thalassemia, Laparotomy closure, Transplantation

## Abstract

Prophylactic augmentation of the wound with mesh proposed by Kniepeiss et al is the world's first attempt to significantly reduce the risk of postoperative hernia in liver transplantation. Similar technique have been described 17 years ago in bariatric patients and confirmed by many studies in various clinical settings. The results of mesh hernia repair in patients on immunosuppressive therapy are not inferior from the data obtained from non- transplant surgery registers.

To reduce the risk of using the mesh in patients scheduled for liver transplantation authors chose absorbable mesh, that maintains the mechanical strength of the wound for up to 18 months. Half of the incisional hernias have been diagnoses more than 3 years from the original procedure.

For prevention of incisional hernias, there is no evidence to support the use of biologic/biosynthetic meshes.

Dear Editor

I read the article by Kniepeiss et al. with great interest. It describes the first attempt to significantly reduce the rate of postoperative hernia in patients who undergo liver transplantation [1]. The technique of primary wound augmentation with the use of mesh was first described in 2002 in a group of patients subjected to a bariatric procedure [2]. Four years later, the results of the first randomized clinical trial of hernia prophylaxis were published [3]. The effectiveness of laparotomy closure, with the use of a non-absorbable mesh, in reduction of the rate of incisional hernia has been confirmed by many studies, among them a multicenter, double-blind, randomized controlled trial by Jairam et al. [4].

The authors of the article published in *Trials* emphasize the risk of using the mesh in patients undergoing immunosuppressive therapy. A large study comparing the use of a mesh in repairing an incisional hernia in patients who underwent liver transplantation or pancreatoduodenectomy showed similar results in both groups, although only patients with transplanted liver were receiving immunosuppressive therapy [5].

According to data obtained from the Americas Hernia Society Quality Collaborative, immunosuppression in patients subjected to open elective ventral hernia repair is associated with an increased risk of 30-day surgical site occurrence, mostly seromas, but not surgical site infection or an additional 30-day morbidity or mortality [6].

To reduce the risk of using the mesh in immunosuppressed patients, the authors chose a long-absorbing mesh, which maintains the mechanical strength of the wound for up to 18 months. Various studies have shown that postoperative hernia is a lifelong risk. Juvany and colleagues have found that, in half of the patients who developed incisional hernia, it occurred more than 3 years from the original procedure [7]. Kockerling et al. proved that in a complex abdominal hernia repair, biologic and biosynthetic meshes do not provide a superior alternative to synthetic meshes [8]. The use of poly-4-hydroxybutyrate mesh to repair incisional hernia in a high-risk group of patients, resulted in a 9% recurrence rate in the 18-month follow-up [9]. There is no evidence to support the use of biologic/biosynthetic meshes for prevention of incisional hernias [8].

I am afraid that the choice of an absorbable mesh may reduce the potential success rate of incisional hernia prevention in patients who undergo liver transplantation.

Yours sincerely,

Janusz Strzelczyk, MD, PhD.

## Data Availability

Not applicable.
